# Teleassessments of Lower Limb Function in Adult Stroke Survivors: A Preliminary Study Evaluating Safety, Feasibility, and Validity for Telerehabilitation

**DOI:** 10.1089/tmr.2024.0052

**Published:** 2024-11-26

**Authors:** Shravni Deshmukh, Sally Freels, Sangeetha Madhavan

**Affiliations:** ^1^ Brain Plasticity Laboratory, Department of Physical Therapy, College of Applied Health Sciences, University of Illinois at Chicago, Chicago, Illinois, USA.; ^2^ Graduate Program in Rehabilitation Science, College of Applied Health Sciences, University of Illinois at Chicago, Chicago, Illinois, USA.; ^3^ Department of Epidemiology and Biostatistics, School of Public Health, University of Illinois at Chicago, Chicago, Illinois, USA.

**Keywords:** teleassessments, remote assessments, stroke, lower limb, telerehabilitation, telehealth

## Abstract

**Introduction:**

Lower limb function teleassessments in adult stroke survivors are critical due to the rise of telerehabilitation. This preliminary study aims to establish the safety, feasibility, and validity of teleassessments, exploring their predictive capacity for walking speed.

**Methods:**

In this cross-sectional study, 17 chronic stroke participants (11 males, 6 females; mean age: 63 ± 8) underwent lab-based and tele-based assessment sessions. Outcome measures included the 30-second chair stand test (30CST), five times sit-to-stand (5XSTS), and 2-minute step test (TMST). 10-Meter walk test categorized participants into slow versus fast (below/above 0.8 m/s). Absolute agreement and bias analyses determined teleassessment validity, while sensitivity and specificity analyses evaluated their effectiveness in classifying slow versus fast walkers.

**Results:**

Absolute agreements were good for 30CST (ICC = 0.885), poor for 5XSTS (ICC = 0.148), and excellent for TMST (ICC = 0.971). 30CST (*p* = 0.8), 5XSTS (*p* = 0.3), and TMST (*p* = 0.2) showed no systematic bias. Similarly, 30CST (*R*^2^ = 0.07, *p* = 0.25), 5XSTS (*R*^2^ = 0.04, *p* = 0.39), and TMST (*R*^2^ = 0.0008, *p* = 0.9) indicated no proportional bias. The TMST demonstrated higher sensitivity (80%) and specificity (50%) compared with 30CST (sensitivity 88% and specificity 12%) and 5XSTS (sensitivity 80% and specificity 25%).

**Discussion:**

30CST and TMST demonstrated adaptability for remote assessment via videoconferencing, establishing their safety, feasibility, and validity. These findings advocate for integrating teleassessments into adult stroke survivors’ telerehabilitation programs.

## Introduction

Telehealth, an evolving tool in stroke care, encompasses stroke telerehabilitation—a promising alternative to in-person therapy.^1–3^ The COVID-19 pandemic accelerated its use to address stroke recovery aspects like motor function, quality of life, activities of daily living, and psychological well-being.^4–8^ Telerehabilitation’s rapid adoption was driven by its potential to overcome barriers such as limited mobility, transportation challenges, provider shortages, and cost-efficiency, thus enhancing access to rehabilitation services.^9,10^ Telehealth’s scope also includes teleassessments, which remotely evaluate progress and treatment needs without in-person visits for motor and behavioral health assessments.^11^

Lower limb function teleassessments have proven safe for individuals with conditions such as multiple sclerosis, spinal cord injury, spina bifida, arthritis, and other mobility disabilities, offering a practical alternative to in-person clinical visits.^12,13^ However, in stroke recovery, where > 75% of survivors experience gait and balance impairments, a significant knowledge gap exists in implementing teleassessments for lower limb function.^14,15^ Current assessments for gait and balance impairments in stroke are typically conducted in person, underscoring the need to adapt established protocols for remote use or develop accurate alternative assessments.^16^

Teleassessments serve as vital clinical benchmarks at every stage of stroke telerehabilitation. Safety, particularly fall occurrence, is a key concern in remote lower limb assessments. Hence, in this study, we selected simple, expert-recommended assessments that require no specialized training or equipment and can be safely administered remotely.^16^ Our primary objective was to evaluate the safety, feasibility, and concurrent validity of select lower limb teleassessments in adult stroke survivors. Our secondary objective was to assess their sensitivity and specificity in predicting walking speed, a significant predictor of post-stroke function.^17^ Most gait-based interventional studies in stroke use walking speed as the primary outcome. However, adapting this measure for remote assessments poses a fall risk and may only be suitable for high-functioning, independent walkers. Therefore, understanding the predictive ability of these teleassessments can provide valuable insights into their use as a surrogate for walking speed measurements.

## Methods

### Study design

This cross-sectional study involved two testing sessions: virtual and in-person, randomized using block randomization. A minimum of 1 week separated the testing sessions to minimize potential bias from task similarity. Persons with chronic stroke were recruited between September 2022 and May 2023. Written informed consent was obtained, and the study was approved by the Institutional Review Board at the University of Illinois at Chicago.

A power analysis using G*Power3 indicated that 14 participants were needed to achieve a power of 0.8 at an alpha level set at 0.05.^18^

### Participants

Inclusion criteria included monohemispheric stroke >3 months, over 21 years old, and Mini MoCA score of >21, ensuring participants’ ability to follow testing instructions. Virtual assessment session requirements included having another adult present for safety, and access to a functioning device with a microphone, speaker, and camera for videoconferencing. Exclusion criteria included significant visual impairment, history of dizziness or angina during physical activity, event(s) of cardiorespiratory disease or renal failure in the past six months, significant cognitive or communication impairments, and lesions involving the cerebellum and the brainstem. Initial participant contact was by telephone, and their consent was obtained on inclusion. Those with a virtual first session remotely signed consent via REDCap, while those with an in-person first session signed in the laboratory.

### Telephone screening

Eligibility was assessed via phone, including a medical screening questionnaire, Mini MoCA administration, a technical aptitude checklist, and balance confidence questionnaires. Following eligibility confirmation after the phone screening, participants were randomly assigned using block randomization to ensure equal group sizes. They either completed teleassessments via videoconferencing followed by lab-based assessments or the reverse order.

### Telephone screening procedures

#### Medical screening questionnaire

Collected data on age, gender, race, medical history, medications, and stroke-related details, including side, type, and time since occurrence.

#### Mini-MoCA scale

A shorter version of the MoCA test, this scale is tailored for easy telephone or video administration, assessing memory and executive function.^19^ Participants scoring below 21 were excluded due to potential comprehension issues compromising their safety.^20^

#### Technical aptitude checklist

This test aimed to assess participants’ familiarity with ZoomPHI, the videoconferencing platform for virtual assessments. Participants were asked about their comfort level with the following: (1) turning on and logging into their device, (2) installing Zoom, (3) enabling the camera and audio, (4) launching Zoom, and (5) checking and sending emails. For participants needing additional support, we arranged an additional session for proficiency in operating their device and utilizing ZoomPHI effectively, ensuring seamless study participation.

#### Activities of Balance Confidence Questionnaire

This self-reported scale comprises 16 items that rate an individual’s perception of balance confidence during everyday activities. Scores range from 0 to 100, with higher scores indicating greater balance confidence. In stroke, the activities of balance confidence (ABC) questionnaire demonstrates excellent test–retest reliability and excellent internal consistency.^21,22^ In addition, it has been successfully administered in virtual settings with stroke patients to evaluate fall risk.^23,24^

#### Stay independent questionnaire

This scale, developed by the U.S. Centers for Disease Control and Prevention’s National Center for Injury Prevention and Control, is a common fall risk screening tool.^25^ A score of 4 points or higher indicates increased fall risk. It was administered alongside the ABC questionnaire as an additional precaution.

### Lower limb functional assessments

A single assessor, a physical therapist, administered both in-person and teleassessment sessions. The following tests were conducted during both sessions:

#### 30-second chair stand test

Participants performed as many sit-to-stand movements as possible in 30 seconds, with the total number of stands becoming their score. This test evaluates lower extremity strength, endurance, mobility, and transfer ability and predicts gait speed and fall risk.^26,27^ It has been safely administered via videoconferencing in lung transplant and knee replacement patients.^28,29^ During the test, participants are instructed to sit in the middle of the chair, with their feet flat on the floor and arms across their chest, then stand and sit repeatedly within the 30-second timeframe, quickly and safely. In the teleassessment session, participants were asked to use a chair without armrests or to avoid armrest use to prevent a score of zero. Caregivers were advised to stand behind the chair for support if needed.

#### Five times sit-to-stand

Participants perform five sit-to-stand movements as quickly as possible, with the total time taken as their score. This test assesses lower extremity strength, mobility, and transfer ability, significantly predicting balance, gait speed, and capacity.^16,29,30^ The five times sit-to-stand (5XSTS) test has been successfully administered virtually in various patient groups, including liver transplant recipients, post-hip surgery patients, and individuals with chronic obstructive pulmonary disease, indicating high acceptance and usability. They also demonstrated the feasibility and strong reliability between clinician-recorded and participant-measured scores in these populations.^30–32^ During the test, participants are instructed to sit in the middle of the chair, feet flat on the floor, arms crossed, and perform five sit-to-stand repetitions. This test was administered simultaneously with the 30-second chair stand test (30CST).

#### Two-minute step test

Participants march in place as fast as possible for 2 min, with the total steps counted as the score. This test evaluates endurance and predicts gait speed and community ambulation.^17,33^ It has been safely conducted virtually with older adults and individuals recovering from COVID-19.^34,35^ Participants lift their knees between knee and hip height for 2 min, with prompts every 30 sec to pace themselves. They can adjust their pace or take short breaks as needed. A sticky note on the wall provides a visual reference for knee-to-hip height. Clear instructions for sticky note placement ensure accuracy during teleassessments. Caregivers provided support if needed, and participants expressing apprehension were given a chair. Arm-supported steps were excluded from the total score.

### Walking speed assessment

This test was administered during the in-person lab session:

#### 10-meter walk test

This test measures walking speed to determine functional mobility, gait, and vestibular function.^36^ Participants performed three trials each of self-selected and maximal walking speeds. Correlations between ambulatory ability and gait speed indicate that increased speed leads to a higher ambulation classification.^37^ We used these classifications to divide participants into two groups: slow walkers (below 0.8 m/s), who may have limited community ambulation, and fast walkers (above 0.8 m/s), who can fully ambulate in the community.

### Teleassessment session additional procedures

Participants and their caregivers received a guide detailing the teleassessment procedures, necessary materials, and emergency contact information. During the session, research personnel shared their Zoom screen displaying visual guides adapted from Jennings et al., 2020 to explain the tests.^38^ Participants were given the opportunity to ask questions. Each assessment began with adjusting the camera angle for optimal visibility of movements for the researcher. A practice repetition was conducted to ensure that participants comprehended instructions and to address any challenges. A technical error log was used to document any errors that occurred during the session, where the error occurred, and what the consequence of that error was.

#### System usability questionnaire

Post study completion, participants completed a qualitative questionnaire rating the teleassessment’s user-friendliness, safety perception, and impact on travel-related inconveniences. We also gathered insights on the clarity of remote instructions, ease of videoconferencing platform use, and preferences between lab-based and teleassessments. Ratings ranged from 1 to 5 with the following interpretation: 1—Strongly Disagree, 2—Disagree, 3 –Somewhat Agree, 4—Agree and 5—Strongly Agree.

#### Safety and feasibility

Safety was assessed by the absence of adverse events (AEs), including pallor, excessive sweating, severe fatigue, dizziness, emotional distress and the occurrence of falls, which were actively monitored during lab and virtual sessions. Participant safety was individually assessed throughout their study involvement. Feasibility was evaluated using compliance, recruitment and adherence metrics. We also documented reasons for study refusals and withdrawals. The technical error form was also factored into study feasibility.

### Statistical analyses

We conducted statistical analyses to assess the safety, feasibility, and concurrent validity of lower limb functional teleassessments in stroke. We focused on examining absolute agreement, systematic bias, and proportional bias. Absolute agreement was assessed using intraclass correlation coefficients (two-way mixed, single measures, i.e., ICC), and interpreted as: poor (<0.5), moderate (0.5–0.75), good (0.75–0.9) and excellent (0.90).^39^ Linear Bland–Altman plots compared lab-based and teleassessment conditions, with linear limits of agreement calculated as mean difference value ±2.12*SD. Systematic bias was tested with one-sample t-tests comparing mean difference values to zero.

Regression-based Bland–Altman plots assessed mean proportional bias using linear regression analysis between difference and mean values. When significant, we plotted the linear regression line with the limits of agreement (linear regression line ± SD of residuals).

Receiver operating characteristic (ROC) curve analysis evaluated the effectiveness of the 30CST, 5XSTS, and 2-minute step test (TMST) teleassessments to classify participants based on walking speeds. The accuracy of each test was quantified using the area under the curve (AUC). AUC values range from 0 to 1 with 1 indicating a perfect test and 0.5 or below suggesting a low diagnostic value. AUC represents the effect of each teleassessment on the probability of correctly classifying participants as slow vs fast walkers. Optimal cut-off points for distinguishing between ambulatory groups were determined by selecting data points that optimized sensitivity and specificity. Statistical tests were performed with SPSS Statistics 27 (IBM, NY, USA), with a statistical significance of *p* < 0.05.

## Results

Participants’ demographic and clinical characteristics are summarized using descriptive statistics in Table 1. Eighteen chronic stroke participants were recruited; 17 completed the study. One individual didn’t participate in assessment sessions due to unresponsiveness after screening.

**Table 1.** Study Participant Characteristics

	Mean (SD)
Age (years)	63 (8)
Sex (male/female)	11/6
Time since stroke (years)	9 (6)
Assistive device for walking (yes/no)	5/12
Mini-MOCA	22 (3)
ABC	72 (17)
Stay Independent Questionnaire	5 (4)
10MWT (slow/fast)	9/8

ABC, Activities of Balance Confidence Questionnaire; SD, standard deviation; 10-MWT, 10-meter walk test.

Note: Values are presented as means and standard deviations; others are categorical values.

### Safety and feasibility

No AEs occurred during both assessment sessions. One technical issue with audio arose during a teleassessment session and was resolved before testing. Teleassessment sessions typically lasted 20–30 min, while in-person sessions lasted 30 to 40 min due to the 10-MWT.

### Validity

#### 30CST

**FIG. 1.**
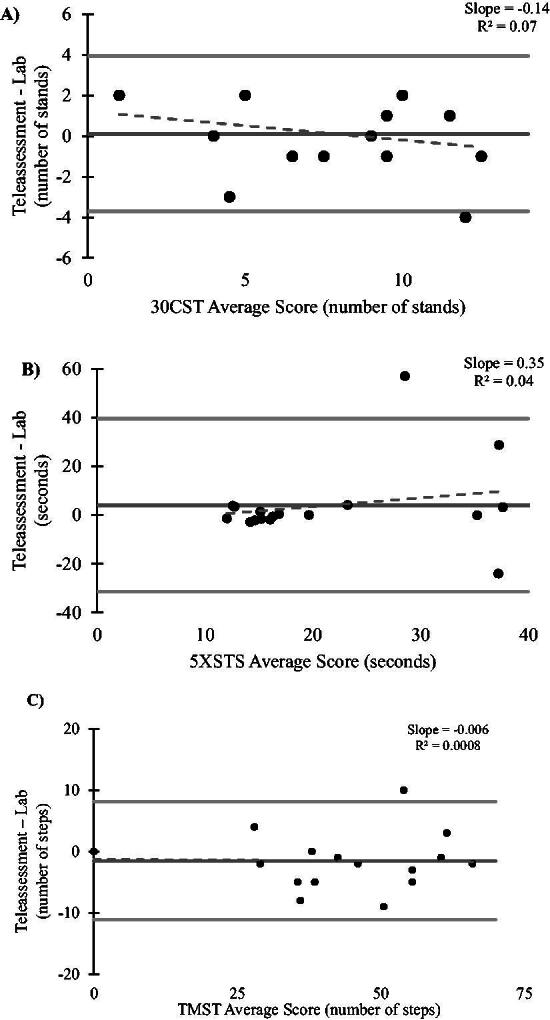
Linear Bland–Altman plots illustrating the relationship between mean and the difference in average scores between teleassessment and lab-based assessment. A) Comparison between 30CST teleassessment and lab assessment; B) comparison between 5XSTS teleassessment and lab assessment; and C) comparison between TMST teleassessment and lab assessment. Horizontal gray lines represent mean difference values (*middle line*) and linear limits of agreement (*upper and lower lines*). Equations and *R*^2^ values for regression lines are displayed. Each data point represents values for one participant. 5XSTS, five times sit-to-stand; 30CST, 30-second chair stand test; TMST, two-minute step test.

High agreement between assessment conditions (ICC = 0.885, 95% CI: 0.712, 0.957), indicating good absolute agreement. Linear Bland–Altman plots revealed no systematic bias (*P* = 0.8) with narrow limits of agreement (3.93, −3.69), suggesting consistent measurements (Fig. 1a). Linear regression analysis showed no proportional bias (*R*^2^ = 0.07, *p* = 0.25). The ROC analyses yielded an AUC of 0.46 ± 0.15, with an optimal cutoff point of 11 stands, resulting in 88% sensitivity and 12% specificity.

#### 5XSTS

Low agreement between assessment conditions (ICC = 0.148, 95% CI: −0.340, 0.575) indicated poor agreement. Linear Bland–Altman plots revealed no systematic bias (*p =* 0.3) with broad limits of agreement (39.43, −31.44), suggesting inconsistent measurements (Fig. 1b). Linear regression analysis showed no proportional bias (*R*^2^ = 0.04, *p* = 0.39). The ROC analyses yielded an AUC of 0.46 ± 0.15, with an optimal cutoff point of 35 sec, resulting in 80% sensitivity and 25% specificity.

#### TMST

High agreement between assessment conditions (ICC = 0.971, 95% CI: 0.923, 0.989) indicated excellent agreement. Linear Bland–Altman plots revealed no systematic bias (*p* = 0.2) with medium limits of agreement (8.07, −11.13), suggesting consistent measurements (Fig. 1c). Linear regression analysis showed no evidence of proportional bias (*R*^2^ = 0.0008, *p* = 0.9). The ROC analyses yielded an AUC of 0.6043 ± 0.15, with an optimal cutoff point of 46 steps, resulting in 80% sensitivity and 50% specificity.

#### System usability questionnaire

Sixteen of 17 participants completed the questionnaire. All participants rated visual and auditory clarity at or above 4, indicating their agreement or strong agreement on a robust audiovisual connection. Ninety percent rated internet connectivity similarly. Eighty-eight percent felt safe during teleassessment sessions. All found the videoconferencing platform easy to use and understood instructions clearly. Fifty percent considered traveling for lab-based assessment burdensome. Eighty-one percent noted no difference between lab-based and teleassessment, with 69% preferring lab-based assessments and 44% preferring teleassessment.

## Discussion

To our knowledge, this is the first study to investigate the safety, feasibility, and concurrent validity of lower limb functional teleassessments in stroke. Teleassessments offer continuous monitoring, enhancing telerehabilitation programs and patient care. All assessments administered virtually in this study proved safe and feasible in persons with stroke. The 30CST and TMST demonstrated good to excellent reliability with low bias, suggesting potential as in-person assessment alternatives. However, 5XSTS demonstrated low reliability, limiting its viability for teleassessment.

The good to excellent reliability of 30CST and TMST aligns with previous studies.^40,41^ However, the low reliability of 5XSTS in our stroke sample contrasts with results from other populations, such as liver transplant recipients and patients with chronic obstructive pulmonary disease (COPD). These studies reported good to excellent agreement between clinician-reported in-person assessments and participant-reported home-based scores.^30,32^ Motor deficits due to neurological impairments seen in stroke are not typically seen in participants with liver transplants and COPD, potentially explaining the discrepancy in results.

ROC curves, with a walking threshold of 0.8 m/s, categorized participants as slow or fast walkers using the 30CST, 5XSTS, and TMST. This threshold, from previous stroke community ambulation studies, served as our reference standard.^37,42^ Participants walking above 0.8 m/s were classified community ambulators with lower fall risk, while those below 0.8 m/s were home and limited community ambulators with higher fall risk. Our findings suggest TMST better discriminates self-selected walking speeds than the 30CST and 5XSTS, which may not be suitable predictors. We determined a TMST cutoff of 46 stands for predicting fast walkers, with a specificity of 50% and sensitivity of 80%. Low specificity indicates a higher potential for false positives. These thresholds may not be clinically relevant.

No AEs occurred during teleassessment sessions, affirming their safety. Rigorous participant screening and an initial practice repetition for each assessment ensured comfort, proper positioning, and a clear view for researchers, minimizing the need for repeated tests due to safety concerns.

Conducting and completing teleassessments were feasible for participants and researchers. Scheduling remote sessions demonstrated advantages over in-person sessions for researchers. Most participants preferred lab-based assessments for social interaction and structured activity despite needing transportation to the lab, many of whom were independent in their daily activities and lived alone. Those favoring teleassessments cited convenience and shorter duration due to elimination of transportation logistics as primary reasons despite needing to coordinate for caregiver supervision. One eligible participant could not enroll as he lived alone and was uncomfortable seeking assistance. Five participants faced challenges coordinating with caregivers due to work or personal commitments. Most participants relied on their significant others for assistance, but some displayed hesitation. Regaining independence in daily activities is a crucial aspect of stroke recovery. In our study, 35% of participants (6 out of 17) lived alone and managed their daily tasks independently; however, we advised them to seek supervision for safety. In future studies, pre-assessing these levels may be necessary to provide appropriate support.

Transitioning from in-person assessments to teleassessments requires considering equipment availability. Participants needed stable chairs for the 30CST and 5XSTS, and caregivers were instructed to stabilize lightweight, plastic chairs to prevent balance issues. For the TMST, sticky notes were necessary; in their absence, paper and tape were used. Informing participants about required materials beforehand helps them prepare for alternatives, ensuring smooth sessions.

Researchers required optimal visibility for accurate assessment scoring. 30CST and 5XSTS required participants to adjust the camera for the visibility of hands, hips, and feet. TMST required the camera to focus on the feet and knees to monitor knee lifting. Device type should also be considered for effective assessment: laptops or tablets provided wider angles, while smartphones often required repositioning.

Study limitations should be considered when interpreting the results. We lacked measures of impairment in our screening, making it hard to determine teleassessment suitability for different stroke severities. Although we obtained a thorough medical history, vital signs such as blood pressure and heart rate could not be assessed remotely, potentially posing safety concerns if medical records were unavailable. Most participants had a higher functional level, as seen on the ABC questionnaire, which limits the study’s representation of the broader stroke population. Those without caregiver access could not enroll, limiting participant inclusivity. This study was conducted in an urban/suburban setting; future research in rural settings may reveal additional barriers. Researchers and clinicians should consider these limitations when interpreting the study’s outcomes.

## Conclusion

The findings of this study demonstrate the adaptability, safety, and feasibility of the 30CST and TMST via videoconferencing for assessing lower limb function in chronic stroke. These findings support their use in telerehabilitation interventions, providing valuable insights for researchers and clinicians aiming to conduct remote synchronous teleassessments in stroke rehabilitation.

## Author Disclosure Statement

The authors declare that there is no conflict of interest.

## Authors' Contributions

The authors declare that there is no conflict of interest. The conception and design of the study: S.D., S.M. Data collection, analyzed the data, and drafted the manuscript for intellectual content: S.D., S.M. Assistance with statistical analysis: S.F. All authors read and approved the final manuscript.

## Data Availability

The data supporting the findings of this study are available on request from the corresponding author.

## Abbreviation Used

ABC questionnaire: Activities of balance confidence questionnaire
AE: Adverse events
AUC: Area under the curve
CI: Confidence interval
ICC: Intraclass correlation coefficient
Mini MoCA: Montreal cognitive assessment 5-minute protocol
ROC curve: Receiver operating characteristic curve
SD: Standard deviation
TMST: 2-minute step test
10MWT: 10-meter walk test
30CST: 30-second chair stand test
5XSTS: Five times sit-to-stand test
